# Expression site of P2RY12 in residential microglial cells in astrocytomas correlates with M1 and M2 marker expression and tumor grade

**DOI:** 10.1186/s40478-016-0405-5

**Published:** 2017-01-10

**Authors:** Changbin Zhu, Johan M. Kros, Marcel van der Weiden, PingPin Zheng, Caroline Cheng, Dana A. M. Mustafa

**Affiliations:** 1 Department of pathology, Erasmus University Medical Center, Wytemaweg 80, 3015 CN Rotterdam, Rotterdam, The Netherlands; 2 Brain center, Erasmus University Medical Center, Wytemaweg 80, 3015 CN Rotterdam, The Netherlands; 3 Division of Experimental Cardiology, Department of Cardiology, Thoraxcenter, Erasmus University Medical Center, Rotterdam, The Netherlands; 4 Department of Nephrology and Hypertension, DIGD, University Medical Center Utrecht, Utrecht, The Netherlands

**Keywords:** Microglia, TAM, Glioma, Glioblastoma, Immunohistochemical markers

## Abstract

The role of resident microglial cells in the pathogenesis and progression of glial tumors is still obscure mainly due to a lack of specific markers. Recently P2RY12, a P2 purinergic receptor, was introduced as a specific marker for microglial cells under normal and pathologic conditions. Here we analyzed the expression of P2RY12 in astrocytomas of various malignancy grades in relation to markers for M1 and M2 macrophage activation profiles by using two web-based glioma datasets and confocal immunohistochemistry to 28 astrocytoma samples grades II-IV. In the gliomas, P2RY12 immunoreactivity delineated CD68 negative cells with otherwise microglial features from CD68 positive tumor associated macrophages (TAMs). The presence of P2RY12 positive cells correlated positively with overall survival. P2RY12 mRNA levels and membrane-bound localization of P2RY12 were inversely correlated with increasing malignancy grade, and the expression site of P2RY12 shifted from cytoplasmic in low-grade gliomas, to nuclear in high-grade tumors. The cytoplasmic expression of P2RY12 was associated with the expression of M1 markers, characteristic of the pro-inflammatory macrophage response. In contrast, the nuclear localization of P2RY12 was predominant in the higher graded tumors and associated with the expression of the M2 marker CD163.

We conclude that P2RY12 is a specific marker for resident microglia in glioma and its expression and localization correspond to tumor grade and predominant stage of M1/M2 immune response.

**Electronic supplementary material:**

The online version of this article (doi:10.1186/s40478-016-0405-5) contains supplementary material, which is available to authorized users.

## Introduction

Microglial cells are brain-specific tissue resident macrophages that are directly derived from yolk-sac erythromyeloid precursor cells (EMP) during embryonic development [19]. As major contributors to the immune status of the central nervous system (CNS) microglial cells scan the CNS for cellular debris by continuously protract and retract their cell processes [26]. The microglial survey mediates the immune response, supports the homeostasis of the neurons and, in collaboration with astrocytes, maintains the integrity of the blood-brain barrier (BBB) [23, 32, 37]. Microglial cells become activated by a large variety of pathogenic situations. Upon activation, the cells take on an amoeboid shape and initiate the paracrine recruitment of blood-derived macrophages into the altered brain parenchyma, initiating an inflammatory response [2, 11]. Microglia serves additional, only partly known, roles in repair processes following the acute stage of tissue damage [18, 27]. Recent studies addressing the phenotypic adaptations of peripheral macrophages in cancer have shown that tumor associated macrophages (TAMs), similar to the M1 and M2 activation states of macrophages, display particular marker profiles of pro- and anti-oncogenic action [25, 31]. TAMs with anti-tumor action share characteristics with M1 macrophages; are capable of antigen presentation and paracrine signaling to promote inflammation, thereby hampering tumor growth and prolonging patient survival [28, 39]. These cells are referred to as M1-like cells. In contrast, TAMs that promote tumor progression are associated with an immunosuppressive response; contribute to tumor angiogenesis and proliferation, and are associated with poor clinical outcomes [10, 17, 33] and these cells are referred to as M2-like cells. In general, TAMs are more similar to M2 - than M1 macrophages [36].

Recent reports have pointed to the heterogeneity of microglial cells [10]. In glioblastoma, microglia displays a pro-oncogenic phenotype resembling that of M2-like macrophages, because the cells are in a microenvironment rich in glioma (stem) cell derived factors like TGF-β1, MCP-1, PGE-2, and soluble colony stimulating factors [22]. There is data showing that resident microglia in glioblastomas plays a role in tumor progression and invasion by the release of cytokines and proteases [24, 35, 38]. However, the mechanism of microglial activation and the contribution of microglia to tumor progression are largely unknown. In order to obtain insight in the specific action of residential microglial cells in gliomas, proper discrimination of these cells from TAMs is necessary. So far, specific markers for residential microglial cells that delineate these cells from other recruited cells of monocyte lineage, are lacking. As a matter of fact, TAMs share many markers with microglial cells. The microglial marker Iba-1 is co-expressed with CD45 and is therefore, not discriminative between residential microglia and monocytic cells that migrated into the brain [13]. In the brains of patients who suffered from Alzheimer disease (AD) the markers CD45 and Iba-1 were used in combination with P2RY12 to separate macrophages from microglia [13]. Considering the growing interest in understanding the role of microglial cells in gliomas, specific markers for the identification of resident microglia in the context of primary brain tumors are urgently needed.

Recently, the Purinergic Receptor P2Y12 (P2RY12) was proposed as a specific marker for healthy rodent CNS microglial cells, discriminating these cells from other types of tissue resident macrophages or blood-derived monocytes [5]. P2RY12 was claimed as a specific marker for microglial cells in human brains [1, 6, 34]. P2RY12 belongs to the family of P2 purinergic receptors, consisting of seven transmembrane G protein coupled receptors (GPCRs) that contribute to ATP-and ADP-mediated cell migration in vitro [7]. P2RY12 is expressed in activated platelets and notoriously, in microglial cells [20]. P2RY12 deficiency in P2RY12 knockdown mice significantly compromised microglial chemotaxis and extension of microglial foot processes in response to CNS injury [12, 29]. In this study, we scrutinized P2RY12 as a marker for microglial cells in glial tumors. We also investigated the relation between the expression of P2RY12 and that of pro- or anti-inflammatory markers; the expression sites in the microglial cells and the relation with tumor progression.

## Material and methods

### Patient samples

All patient samples were obtained from the Archives of the Department of Pathology, Erasmus Medical Center, Rotterdam, with approval of medical ethical committee of the Erasmus Medical Center. Twenty-eight glioma samples (11 astrocytomas WHO grade II (A II), 7 anaplastic astrocytomas (WHO grade III; AA) and 10 glioblastomas (GBMs) were collected and all diagnoses were confirmed by a certified pathologist (JMK). The mean ages ± standard deviations and the male/female ratio are summarized in (Table 1). Four autopsy brains of patients who did not have brain tumors were used as a control. Post-mortem times of the control cases were 8 h or less.

**Table 1:** summary of the patients used for immunohistochemistry

	Astrocytoma grade II (All)	Anaplastic astrocytoma (AA)	Glioblastoma (GBM)	Control (autopsy brains)
Mean age ± st. dev.	42 ± 12.8	40 ± 12.8	45 ± 14.9	58 ± 13.3
Male/Female	4/7	4/3	7/3	2/2

### Immunostaining

Adjacent sections of 5 μm thickness from Formalin Fixed Paraffin Embedded (FFPE) samples were used for immunohistochemical analysis. The sections were incubated with antibodies against P2RY12 (1:100; Sigma, Sweden); CD68 (1:800; Dako, Denmark) GFAP (1:200; Dako, Denmark); CD45 (1:100; Dako, Denmark) and CD163 (1:400; Abd Serotec, USA). The staining procedure and scanning of the stained sections were performed according to the protocol described previously [40]. For double labeling, Alexa Fluor 488 and 555-conjugated secondary antibodies (1:200, Thermo Fisher Scientific, The Netherlands) were used for detection. For triple staining, Goat anti-Mouse F(ab)2 fragment (Jackson ImmunoResearch, WestGrove, PA, USA) was used to block background epitopes and Alexa Fluor 647-conjugated secondary antibody (Thermo Fisher Scientific, The Netherlands) was used for detection. The fluorescent labeled samples were analyzed by using the confocal microscope LSM 700 (Zeiss, The Netherlands). Signal positive areas and staining intensity were quantified using the Image J program to five high power field (40x) areas of each immunostained slide.

### Public database

The transcription level of P2RY12 was assessed in human gliomas of various WHO grades, using 2 different datasets derived from the public NCBI GEO database (GDS 4467 Gene ID 64805 and GDS1816 Gene ID 64805). In addition, transcriptional data of P2ry12 in a murine glioma model were obtained from the GEO database (GSE 86573) [3].

RNA-sequencing data of P2RY12 combined with clinical data were obtained from two TCGA glioblastoma databases (Provisional: 166 GBM patients [4]. In addition, information on IDH1 mutation as well as MGMT methylation status were also obtained from the TCGA GBM database (Cell, 2013). The R program and CGDS-R package provided by the cBioPortal for cancer genomics from Memorial Sloan-Kettering Cancer Center was used to download and process data.

To study the relation between P2RY12 mRNA expression levels and markers for microglia identification, a mutual exclusivity analysis was performed as described previously [8, 9]. The GBM TCGA dataset (GBM, provisional) and a low-grade glioma TCGA dataset were selected for analysis using the cBioPortal websource (Additional file 1: Table S1). High P2RY12 expression was defined by a Z score ≥ 2. Z score = (individual P2RY12 value – mean P2RY12 value) / std. dev. of the whole sample set. A log Odd Ratio (OR) >0 indicates a trend of co-occurrence in expression of P2RY12 with the listed genes and log (OR) <0 indicates a trend of mutual exclusivity in expression pattern.

### Statistics

The results of immunostaining and the data from public database were analyzed by the Mann-Whitney *U* test (*P* < 0.05 was considered significant). Survival analysis was performed using the Log-rank test (*P* < 0.05 was considered significant). All data and graphs were analyzed and made by using Graph pad Prism 5.0.

## Results

### Reduction of membrane P2RY12 signal correlates with glioma grade

**Fig. 1:**
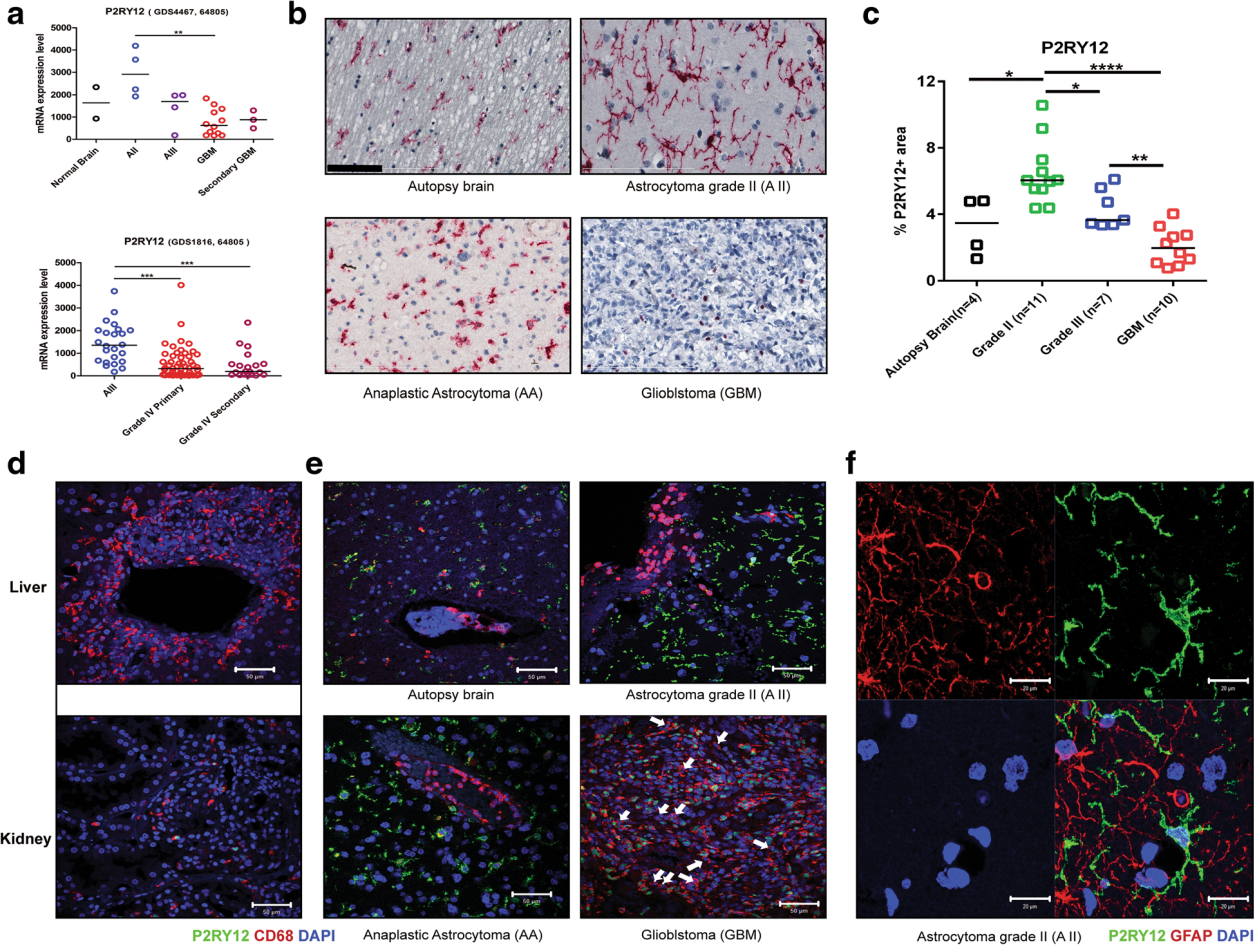
Reduced P2RY12 expression and nucleus translocation in microglia/macrophages correlated with glioma progression. **a**: Analysis of GEO databases reveals decrease of P2RY12 mRNA expression with progression of glioma. Results are shown in the median values of P2RY12 mRNA levels. ** *P* < 0.01; ***P < 0.005. A II: Astrocytoma WHO grade II, AA: Anaplastic Astrocytoma (WHO grade III), GBM: Glioblastoma. **b**: Immunohistochemical staining for P2RY12 in normal brain and different grades of glioma. Representative pictures were selected from each group. Cells with the P2RY12 signal localized mainly in the cytoplasm display the classic ramified microglial morphology in autopsy brain, A II and AA. Cells with nuclear location of P2RY12 are mainly observed in GBM samples (scale bar: 100 μm). **c**: Quantification of percentages of P2RY12 positive areas per image field in autopsy brain, A II and GBM. Mean values of each group are indicated. * *P* < 0.05, ** *P* < 0.01 ****P < 0.001. **d**: Confocal images showing CD68 and P2RY12 staining in tissue samples of liver and kidney (scale bar: 50 μm). **e**: Confocal images showing the spatial relation between CD68 and P2RY12 positive cells in autopsy brain, A II, AA and GBM (scale bar: 50 μm). **f**: Confocal images showing no overlap between GFAP and P2RY12 staining in astrocytoma grade II (scale bar: 20 μm)

The analysis of the public databases revealed that P2RY12 is mainly expressed in A II, while less in AA and GBM (Fig. 1a). The results of immunostaining for P2RY12 are shown in Fig. 1b. P2RY12 positive cells in autopsy brains and A II presented the typical ramified morphology with extended processes. In contrast, in AA a mixture of both ramified and amoeboid P2RY12 positive cells were noticed. In the GBMs the P2RY12 staining was less on the cell membranes while the signal was mainly visible in the nuclei (P2RY12^nuclei+^). Quantitative analysis of the percentage of P2RY12 positive areas per image view revealed a significant smaller number of P2RY12 positive cells in the AA and GBM as compared to A II (Fig. 1b, c). Double staining of P2RY12 with the pan- macrophage marker CD68 revealed a large population of CD68 negative, P2RY12 positive cells (CD68^-^ P2RY12^+^) distinct from TAMs (CD68^+^ P2RY12^-^) in all glioma samples (Fig. 1e). The population of P2RY12^+^ cells did not overlap with the population of GFAP positive tumor cells or reactive astrocytes (Fig. 1f). In autopsy brain, A II and AA, the CD68^+^ TAMs generally had round cell bodies and were located around the blood vessels. In GBM, CD68^+^ TAMs were not only located around the blood vessels (Fig. 1e, white arrows), but were spatially mixed with the P2RY12^nuclei+^ cells.

### P2RY12^nuclei+^ cells are not of myeloid origin, but represent resident microglia

**Fig. 2:**
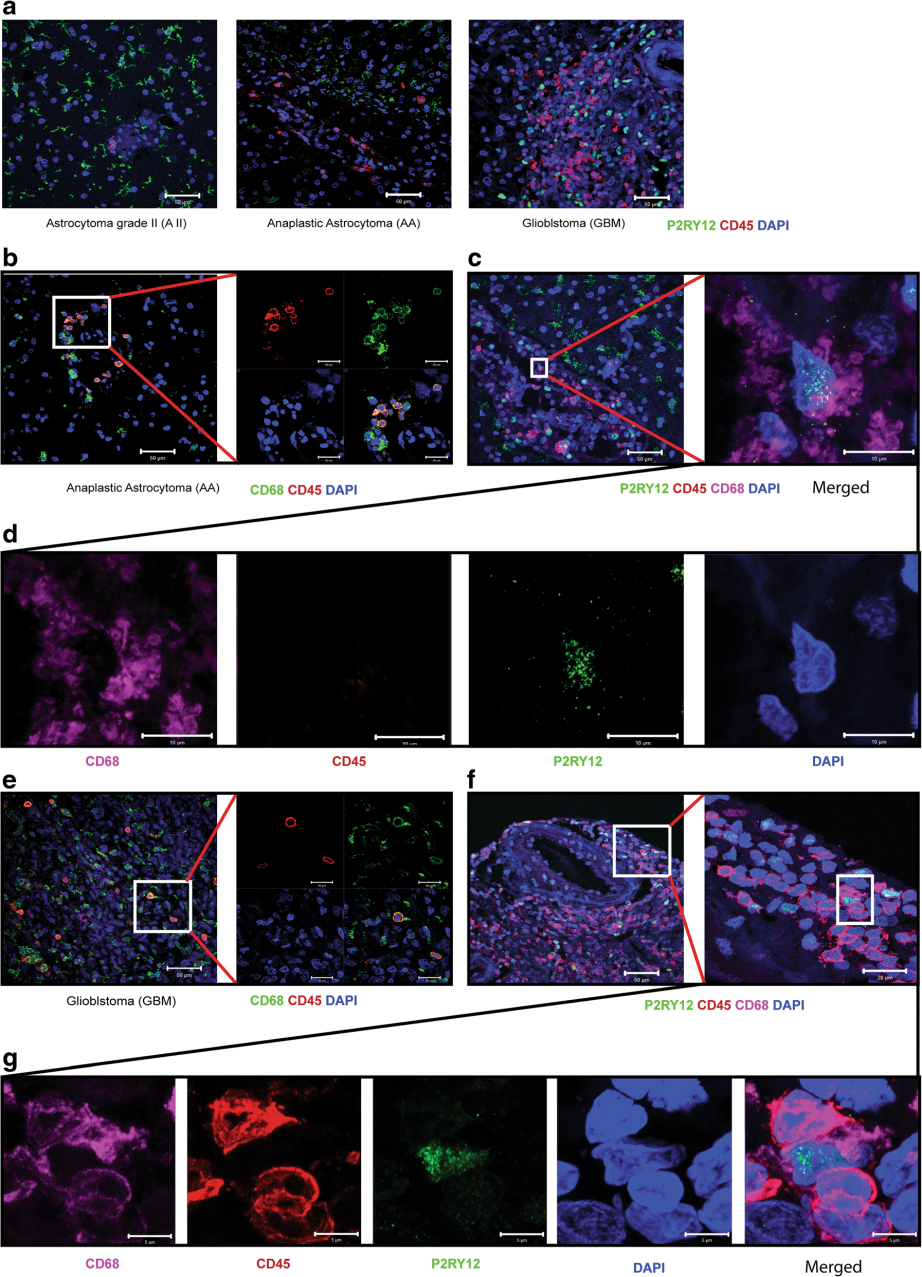
Cells in high grade glioma expressing P2RY12 in the nuclei are not recruited cells of myeloid origin, but resident microglia. **a**: Confocal images showing P2RY12 and CD45 signals in A II, AA and GBM. No overlap between these two markers was observed in any of the gliomas (scale bar: 50 μm). **b**: Confocal images showing CD68 and CD45 signals in AA (scale bars: *left* image; 50 μm, *right* panel; 20 μm). **c**: Confocal images of triple staining for CD68, P2RY12 and CD45 in AA. *Left* image: Merged overview of triple staining (scale bar: 50 μm). *Right* image: Merged high magnification of a P2RY12 positive cell (scale bar: 10 μm). **d**: Panel of single channel images of a P2RY12 positive cell in AA (scale bar: 10 μm). **e**: Confocal images showing CD68 and CD45 staining in GBM (scale bar: *left* image; 50 μm, *right* panel; 20 μm). **f**: Confocal images showing triple staining for CD68, P2RY12 and CD45 in GBM. *Left* panel: Merged overview of triple staining (scale bar: 50 μm). *Right* panel: Merged higher magnification view of P2RY12 positive cells (scale bar: 10 μm). **g**: Panel of high magnification single channel images of P2RY12 positive cells in GBM (scale bar: 10 μm)

Higher numbers of CD45 positive cells were observed in AA and GBM than in A II. The CD45+ cells were mostly present around the blood vessels (Fig. 2a, A II versus AA versus GBM). Double staining for CD45 and P2RY12 did not reveal double-stained cells, indicating that the P2RY12 positive cells are not derived from myeloid lineage (Fig. 2a). CD45 positive cells were observed in higher graded tumors, prominently around blood vessels (Fig. 2a, A II versus AA versus GBM). To further evaluate the identity of CD68^+^P2RY12^nuclei+^ cells double immunostaining for CD45 and CD68 (Fig. 2b and e) and triple immunostaining for CD68, CD45 and P2RY12 was carried out. Two large cell populations were observed, namely CD68^+^CD45^+^P2RY12^-^ and CD68^+^CD45^-^P2RY12^nuclei+^ cells, the former to be interpreted as monocytes/macrophages, and the latter as the resident microglial population (Fig. 2c, d, f, g).

In the GEO dataset GSE 86573 generated from a murine glioma model the expression of P2ry12 by microglia (MG) was significantly higher than that by bone marrow- derived macrophages (BMDM) (Additional file 2: Figure S1).

### Cytoplasmic or nuclear distribution of P2RY12 in microglia relates to the functional marker profiles

**Fig. 3:**
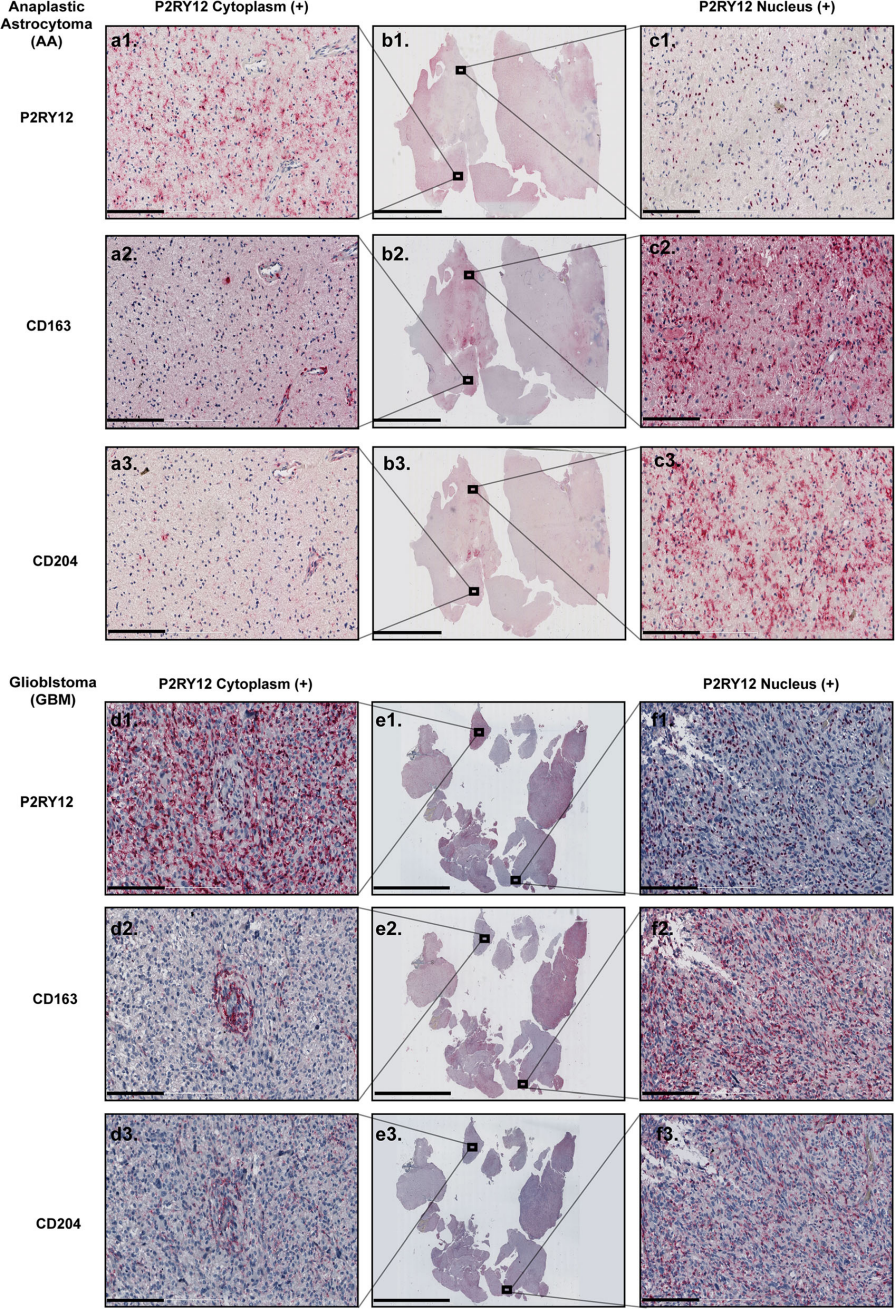
Cytoplasmatic and nuclear distribution of P2RY12 in microglia is correlated with M2-like activation in high grade glioma. **a1**-**a3**: Co-expression of cytoplasmic P2RY12 with CD163 and CD204 in AA (scale bar: 200 μm). **b1**-**b3**: Overview of AA tissue samples (scale bar: 10 mm). **c1**-**c3**: Co-expression of nuclear P2RY12 with CD163 and CD204 in AA (scale bar: 200 μm). **d1**-**d3**: Co-expression of cytoplasmic P2RY12 with CD163 and CD204 in GBM (scale bar: 200 μm). **e1**-**e3**: Overview of GBM tissue samples (scale bar: 10 mm). **f1**-**f3**: Co-expression of nuclear P2RY12 with CD163 and CD204 in GBM (scale bar: 200 μm)

**Fig. 4:**
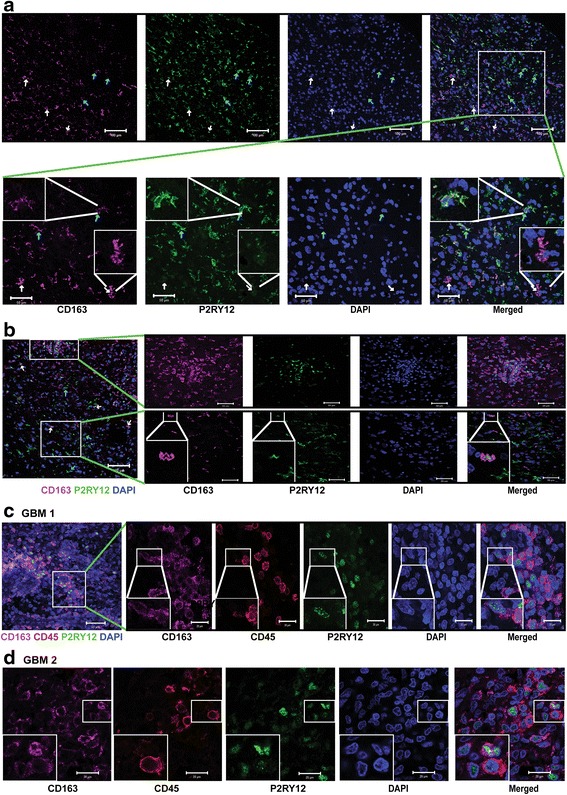
Confocal analysis of co-localization of P2RY12, CD163 and CD45 in GBM. **a**: *Upper row*: overview of immunostaining for P2RY12 and CD163 in a representative GBM. *White arrows* indicate nuclear P2RY12 signal in cells with high CD163 signal; Cyan *arrows* indicate cytoplasmic P2RY12 signal in cells with low CD163 signal (scale bar:100 μm). *Lower row*: selected view of P2RY12 and CD163 staining in glioblastoma. *White arrows* indicate cells with weak P2RY12 and high CD163 signal (scale bar: 50 μm). Inserts: details of cells with low CD163 and strong cytoplasmic P2RY12 signals, and cells with strong CD163 and weak P2RY12 signals. **b**: Overview with two inserts. *Upper* panel: area with cells with nuclear P2RY12 signal with strong CD163 expression (scale bar: 50 μm). *Lower* panel: area with cytoplasmic P2RY12 signals and weak CD163 expression (scale bar: 50 μm). *White arrows*: nuclear P2RY12 expression with strong CD163 signals. **c**: Overview: triple staining for CD163, P2RY12 and CD45 in GBM (scale bar: 50 μm). Inserts (scale bar: 20 μm): Cells with high CD163 and nuclear P2RY12 signals did not overlap with CD45. **d**: Confocal images showing triple staining for CD163, P2RY12 and CD45 in GBM (scale bar: 20 μm). Cells with high CD163 and nuclear P2RY12 signal did not overlap with CD45

The results of immunostaining for the M2 markers CD163, CD204 and P2RY12 showed that cytoplasmic P2RY12 expression did not overlap with CD163 and CD204 positivity (Fig. 3 a1-3, d1-3). In contrast, cells with nuclear located P2RY12 showed distinct overlap with CD163 and CD204 (Fig. 3 c1-3, f1-3). Confocal analysis revealed that cells with a low CD163 signal show ramified cell processes and higher P2RY12 signals. Cells with a high CD163 signal are either negative for P2RY12, or display nuclear localization of P2RY12 (Fig. 4a and b, cyan and white arrows). Triple labeling for CD45, CD163 and P2RY12 differentiated between two cell populations: CD163^+^ P2RY12^nuclei+^ cells devoid of CD45 signals and CD163^+^ CD45^+^ P2RY12^+^ cells (Fig. 4c and d).

### High expression levels of P2RY12 are associated with favorable clinical outcomes

**Fig. 5:**
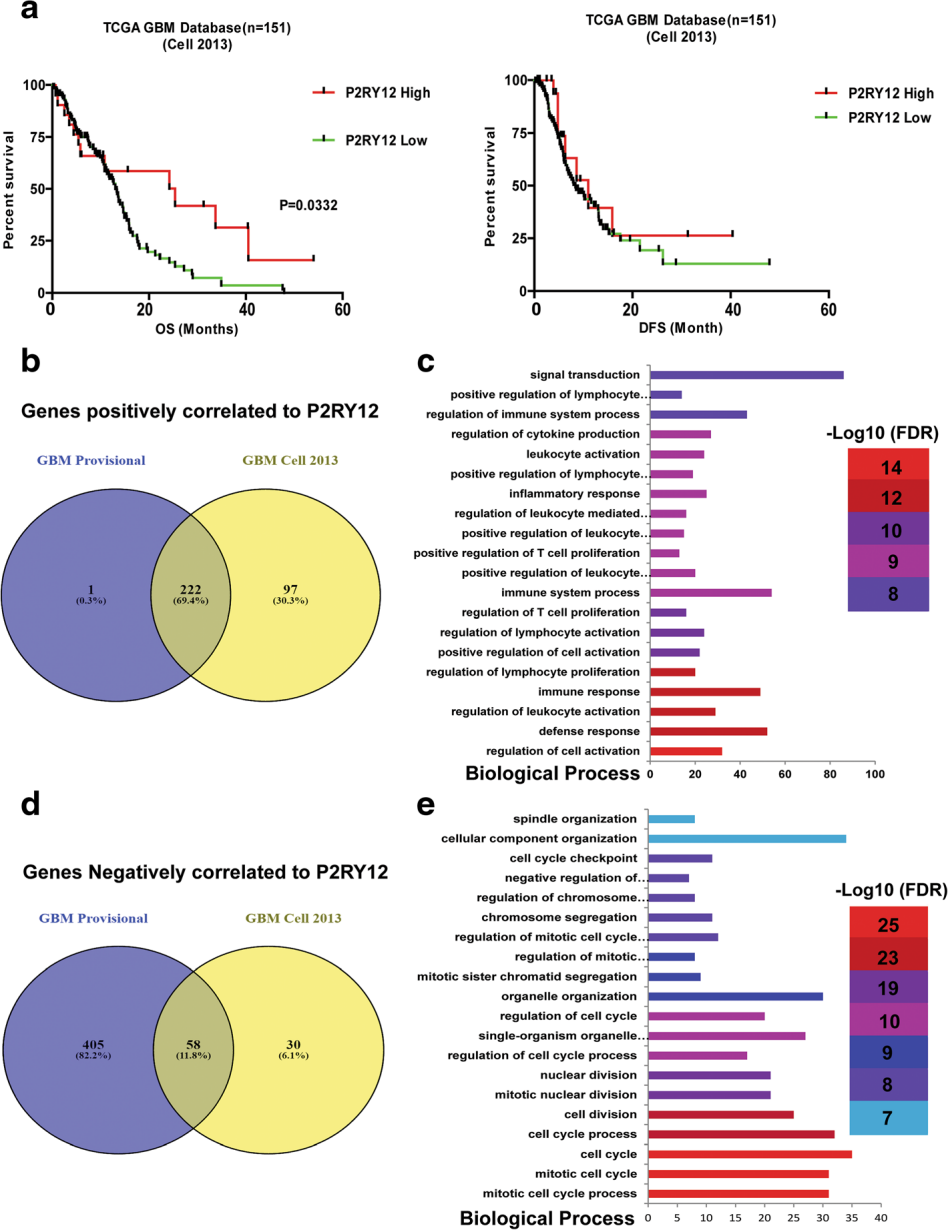
Analysis of TCGA datasets: High expression levels of P2RY12 in GBM patients predict a favorable outcome. **a**: Kaplan-Meier curves showing disease free survival and overall survival curves of patients with different P2RY12 expression levels. **b**: Overlap of the genes found in the two datasets that positively correlate with P2RY12 expression. **c**: Top 20 most significant biological processes resulting from functional annotation of the 222 overlapping genes. The GO terms are shown on the y-axis. The numbers of input genes per GO term are indicated on the x-axis. **d**: Overlap of the genes found in the two datasets that negatively correlate with P2RY12 expression. **e**: Top 20 most significant biological processes resulting from functional annotation of the 58 overlapping genes. The GO terms are shown on the y-axis. The numbers of input genes per GO term are indicated on the x-axis

P2RY12 RNA expression data was obtained from two TCGA glioma databases. From the glioma database [4], 151 patients were collected for analysis (Table 2). Twenty-one tumors with at least 1-fold increase of P2RY12 expression were defined as the “P2RY12 high” group, while the tumors expressing P2RY12 al lower levels than 1-fold increase were defined as the “P2RY12-low” group. Patients with tumors of the P2RY12-high group had longer overall survival times (*P* = 0.03), but not a longer disease free survival than those in the P2RY12-low group (Fig. 5a). In the glioma provisional database from TCGA the P2RY12-high group showed a trend of longer disease free survival (*P* = 0.0608) as well as a significant increase in overall survival time (*P* = 0.0446) (Additional file 3: Figure S2a and b, upper and lower graph). Univariate analysis by the Log-rank test revealed that patients in the P2RY12-high group have lower hazard ratios for disease recurrence (HR = 0.445, *P* = 0.0187) than patients in the P2RY12 low group. Similarly, the P2RY12-high group has a lower hazard ratio for overall survival versus the P2RY12-low group (HR = 0.4598, *P* = 0.04) (Additional file 3: Figure S2c). The data show that P2RY12 up-regulation positively correlates with prognosis and overall survival of GBM patients. The expressional levels of P2RY12 are significantly higher in gliomas with the IDH1 (R132H) mutation as compared to the IDH wt gliomas (Additional file 4: Figure S3a). There is, however, no effect of P2RY12 expression on overall survival when the population was stratified for IDH1 mutation status (Additional file 4: Figures S3b and c). There were no expressional differences of P2RY12 between gliomas with or without MGMT promoter methylation (Additional file 5: Figure S4a). The expression of P2RY12 did not affect the overall survival of the patients when stratified for MGMT promoter methylation status (Additional file 5: Figures S4b and c).

**Table 2:** Summary of patients’ information from TCGA GBM database

TCGA GBM Database *n* = 151			
P2RY12		High (*n* = 21)	Low (*n* = 130)
mRNA level ± S.D	(RNA Seq V2 RSEM)	3263 ± 722.6	499.5 ± 580.1
Age (year) ± S.D		55.1 ± 15.1	61.5 ± 12.4
Gender	Male	17(21)	79(130)
	Female	4(21)	51(130)
IDH1 mutation	WT	17(21)	127(130)
	Mutant	4(21)	3(130)
Subtype	Neural	7(21)	19(130)
	Pro-Neural	1(21)	27(130)
	Classical	4(21)	35(130)
	Mesenchymal	5(21)	44(130)
	G-CIMP	4(21)	4(130)
	N/A	0	1(130)
Therapy	TMZ Chemoradiation, TMZ Chemo	9(21)	55(130)
	Standard Radiation, TMZ Chemo	3(21)	21(130)
	Nonstandard Radiation, TMZ Chemo	2(21)	11(130)
	Standard Radiation, Alkylating Chemo	1(21)	
	Unspecified Radiation	6(21)	17(130)
	Alkylating Chemo		1(130)
	Standard Radiation		12(130)
	Standard Radiation, Alkylating Chemo		3(130)
	TMZ Chemo		1(130)
	Nonstandard Radiation		4(130)
	Unspecified Therapy		5(130)

### P2RY12 expression correlates with microglia/macrophage function

P2RY12 had a significant likelihood for co-occurrence with the microglia markers Iba-1 and CX3CR1 in GBM, and CX3CR1, CD11b in low grade glioma. Likewise, IRF8, a transcription factor for the function and development of microglia cells [14, 15] is expressed simultaneous with P2RY12 in both GBM and low grade gliomas. Due to the limited number of samples in the high P2RY12 expression group, the p-values of associations with log(OR) < -3 remain non-significant, despite a high probability and tendency for mutual exclusion.

The associations between P2RY12 and other markers are listed in the Additional file 1: Table S1. Genes positively (Spearman r ≥0.5) or negatively (Spearman r ≤ -0.3) correlated with P2RY12 from the two TCGA GBM database were selected for pathway enrichment analysis (Fig. 5b, d). Pathway analysis from Gene Ontology based on 222 P2RY12 positively correlating genes included the regulation of immune response including defense response, leukocyte activation, T cell activation (Fig. 5c). Pathway analysis on 58 P2RY12 negatively correlating genes mainly included cell cycle regulation and cell proliferation (Fig. 5e). The findings indicate that P2RY12 expression is positively correlated with markers commonly used for microglia in the CNS and positive immune response, but is inversely associated with markers for peripheral recruited macrophages, M2-like microglia/macrophage activation and cell proliferation.

## Discussion

In the present study, we found that resident microglial cells in gliomas specifically express P2RY12 and that the expression distinguishes microglia from other monocytes and macrophages. This finding in human gliomas corroborates the data we generated from the murine gliomas represented in the GEO database. The analysis of the public glioma datasets and the multi-labeling experiments of the glioma biopsy specimens confirmed that P2RY12 mRNA and protein expression is confined to resident microglial cells. In addition, P2RY12 expression is associated with tumor grade: the expression is less in AA and GBM, as compared to A II. However, the expression of P2RY12 is not an independent prognosticator in gliomas; when strong prognostic factors as IDH mutational status or methylation status of MGMT are taken into consideration, no additional effects of the expression are found. The expression of P2RY12 appeared to be higher in the IDH mutated tumors, which is in line with the association of IDH mutation and better prognosis on the one hand, and the pro-inflammatory status on the other. Remarkably, with increasing malignancy grade there is a shift from cytoplasmic to nuclear expression, and in the high-grade tumors the nuclear expression of P2RY12 coincides with that of the M2 markers CD163 and CD204 (Fig. 1a and c, Fig. 4). We also observed that in the high-grade gliomas the P2RY12 positive microglial cells have taken an amoeboid phenotype (Fig. 1b).

Upon pathologic stimuli, resting microglia adopt a highly dynamic phenotype referred to as “ramified microglia”, with an extensive motile set of foot processes that continuously survey the local environment to recognize and eliminate pathogens [6]. Loss of microglial expression of P2RY12 in knockout mice resulted in impaired polarization, migration and extension of microglial processes towards extracellular nucleotides released from damaged cells, indicating that P2RY12 is required to guide microglial chemotaxis [12]. Further studies revealed that a raise of local extracellular ATP/ADP levels at the site of CNS injury activates Gi/o-coupled P2RY12, followed by PI3K and PLC signaling-mediated migration of microglial cells towards the chemotactic source [7]. Exogenous stimuli like lipopolysaccharides (LPS) can cause a dramatic reduction of the P2RY12 expression in microglia cells in vitro accompanied by the retraction of microglial processes and metamorphosis into an amoeboid shape [12]. These phenomena indicate a function of P2RY12 in the activation of immune regulation during inflammation.

The environmental changes taking place under various pathological conditions cause ATP/ADP leaks that are noticed by the P2RY12 receptors of microglia and lead to changes affecting the cell processes and motility of the cells [16]. High concentrations of purinergic nucleotides and nucleosides such as adenosine and ATP were shown to work in synergy with LPS activation of microglial cells, promoting chemo repulsion away from the ATP source, a process that is associated with increased local adenosine A2A receptor signaling [16, 30]. The expression of the adenosine A2A receptor increases significantly in response to LPS, while P2RY12 expression decreased by LPS, indicating that the shift from a ramified towards an amoeboid phenotype depends on the balance between P2RY12 and A2A receptor signaling, respectively [16]. More studies are required to elucidate the exact regulatory mechanism of microglia immune-activation by these two significant signaling pathways.

In this study we observed nuclear localization of P2RY12 in microglial cells in the high-grade tumors, while in the lower graded astrocytomas P2RY12 was expressed in the cytoplasm. G protein-coupled receptors (GPCRs), such as P2RY12 and its family members are considered as cell surface bound mediators of intracellular signaling. From in vitro and in vivo studies, it is known that some receptors have a nuclear localization. These receptors include the receptors for apelin, angiotensin II AT1, parathyroid hormone, glutamate mGluR5, endothelin ETA and ETB, and the prostaglandins EP1, EP3, and EP4 [21]. The mechanism and functional implications of nuclear translocation remains obscure. It has been suggested that nuclear import is programmed by the DNA sequence of the receptors. Nuclear GPCRs complex proteins such as heterotrimeric G proteins, phospholipase A2, and phospholipase C seem to remain active in intracellular signaling, similar to the activation of nuclear endothelin and prostaglandin receptors that were proven to increase nuclear Ca^2+^concentrations [21]. However, the functional implications of nuclear localization of the P2RY12 receptor and its association with advanced tumor grade needs to be further unraveled.

## Conclusion

The expression site of P2RY12 matches astrocytoma grade, and also reflects the activation status of microglia cells in the tumors. Because of its association with the stage of immune response, P2RY12 may become an interesting drug target for future immune-modulation based therapy for the patients suffering from these tumors.

## Additional files

Additional file 1: Table S1. Summary of mutual exclusivity analysis from two TCGA glioma database. TCGA GBM provisional database and low grade glioma database were analyzed from cBioPortal. P2RY12 upregulation was defined by Z score. Z score = (Individual P2RY12 value-mean value)/Standard deviation of whole sample set. (DOC 62 kb)
Additional file 2: Figure S1. Higher expression of P2ry12 in microglia than bone marrow derived macrophages in a mouse glioma model. It appears from the GEO dataset (GSE 86573) that P2ry12 mRNA is expressed by microglia (MG) and not by bone marrow-derived macrophages (BMDM) (Results are shown as Mean ± S.E.M representing the level of P2ry12 mRNA); ** *P* < 0.01. (TIF 2273 kb)
Additional file 3: Figure S2. High expression of P2RY12 in GBM patients is predictive of favorable outcome. a: Overview of P2RY12 expression levels of 166 GBM samples from the TCGA GBM provisional database. b: (upper panel) Kaplan-Meier curves showing significantly different disease free survival between patients with high, versus low- or intermediate P2RY12 expression levels. (lower panel) Kaplan-Meier curves showing significantly different overall survival between patients with high, versus low or intermediate P2RY12 expression levels. c: Graph showing hazard ratios (HR) with 95% confidential interval (CI) of disease free survival and overall survival for the distinct P2RY12 expression levels. (TIF 72698 kb)
Additional file 4: Figure S3. Expression of P2RY12 does not influence overall survival in groups stratified for IDH1 mutation status (TCGA database). a. Increased P2RY12 mRNA expression in gliomas with IDH1 (R132H) mutation. Results are shown for the median values of P2RY12 mRNA levels; * *P* < 0.05; WT: IDH Wild type. b. Kaplan-Meier curves showing no effect of P2RY12 expression level on overall survival within the group of patients with IDH1 wild type gliomas (*P* > 0.1). c. Kaplan-Meier curves showing no effect of P2RY12 expression level on overall survival within the group of patients with IDH1 mutant (R132H) gliomas (*P* > 0.1). (TIF 10499 kb)
Additional file 5: Figure S4. P2RY12 does influence overall survival in gliomas stratified for MGMT promoter methylation status (TCGA database). a. P2RY12 mRNA expression levels in gliomas with and without MGMT promoter methylation (median values of P2RY12 mRNA levels are shown). b. Kaplan-Meier curves showing no influence of P2RY12 expression level on overall survival within the glioma group with methylated MGMT promoter. c. Kaplan-Meier curves showing no influence of P2RY12 expression level on overall survival within the glioma group with unmethylated MGMT promoter. (TIF 14397 kb)
