# Supplementary material for: Expression site of P2RY12 in residential microglial cells in astrocytomas correlates with M1 and M2 marker expression and tumor grade
Source: Acta Neuropathol Commun. 2017 Jan 10;5:4. doi: 10.1186/s40478-016-0405-5 (PMC5223388; doi:10.1186/s40478-016-0405-5)
Supplement: Additional file 1: Table S1. — Summary of mutual exclusivity analysis from two TCGA glioma database. TCGA GBM provisional database and low grade glioma database were analyzed from cBioPortal. P2RY12 upregulation was defined by Z score. Z score = (Individual P2RY12 value-mean value)/Standard deviation of whole sample set. (DOC 62 kb) [file 40478_2016_405_MOESM1_ESM.doc]

**Supplementary Table 1. Summary of mutual exclusivity analysis from two TCGA glioma database**

TCGA GBM provisional database and low grade glioma database were analyzed from cBioPortal.

P2RY12 upregulation was defined by Z score. Z score=(Individual P2RY12 value-mean value)/Standard deviation of whole sample set.

| **Mutual Exclusivity to P2RY12 Expression** |  | **TCGA GBM provisional database(RNA-Seq) n=166 Upregulation: Z≥2** | | | **TCGA Low grade glioma provisional database(RNA-Seq) n=530 Upregulation: Z≥2** | | |
| --- | --- | --- | --- | --- | --- | --- | --- |
|  | **Genes** | **Log (OR)** | **P-Value** | **Association** | **Log (OR)** | **P-Value** | **Association** |
| **Microglia/Macrophage markers** | **AIF1 (Iba-1)** | 1.83 | 0.033 | Co-occurrence | 1.1 | 0.11 | NS |
|  | **CX3CR1** | 2.56 | 0.0076 | Co-occurrence | 3.25 | <0.001 | Co-occurrence |
|  | **IRF8** | 3.64 | <0.001 | Co-occurrence | 2.06 | <0.001 | Co-occurrence |
|  | **ITGAM (CD11b)** | 0.861 | 0.861 | NS | 1.81 | 0.01 | Co-occurrence |
| **Recruited myeloid cell markers** | **PTPRC (CD45)** | <-3 | 0.83 | Mutual exclusivity | 1.1 | 0.11 | NS |
|  | **CCR2** | <-3 | 0.6 | Mutual exclusivity | 0.85 | 0.25 | NS |
|  | **ITGAX (CD11c)** | 1.672 | 0.094 | NS | <-3 | 0.28 | Mutual exclusivity |
| **Activated phenotype markers** | **CD163** | <-3 | 0.83 | Mutual exclusivity | <-3 | 0.5 | Mutual exclusivity |
|  | **MSR1 (CD204)** | <-3 | 0.6 | Mutual exclusivity | 0.055 | 0.63 | NS |
|  | **NFKB1** | <-3 | 0.6 | Mutual exclusivity | <-3 | 0.5 | Mutual exclusivity |
|  | **IRF4** | <-3 | 0.68 | Mutual exclusivity | <-3 | 0.91 | Mutual exclusivity |
|  | **STAT1** | 1.95 | 0.026 | Co-occurrence | 0.055 | 0.629 | NS |
|  | **STAT3** | <-3 | 0.685 | Mutual exclusivity | -0.136 | 0.685 | Mutual exclusivity |
|  | **STAT6** | <-3 | 0.528 | Mutual exclusivity | 0.671 | 0.43 | NS |
|  | **NOS-2** | <-3 | 0.68 | Mutual exclusivity | <-3 | 0.66 | Mutual exclusivity |
| **Secreted factors** | **IL1β** | 0.48 | 0.51 | NS | 1.15 | 0.097 | NS |
|  | **IL4** | <-3 | 0.68 | Mutual exclusivity | -0.37 | 0.53 | Mutual exclusivity |
|  | **IL6** | <-3 | 0.25 | Mutual exclusivity | <-3 | 0.87 | Mutual exclusivity |
|  | **IL10** | <-3 | 0.64 | Mutual exclusivity | 0.5 | 0.48 | NS |
|  | **CXCL8** | <-3 | 0.56 | Mutual exclusivity | <-3 | 0.91 | Mutual exclusivity |
|  | **CXCL9** | <-3 | 0.56 | Mutual exclusivity | 0.88 | 0.37 | NS |
|  | **CXCL10** | <-3 | 0.56 | Mutual exclusivity | <-3 | 0.45 | Mutual exclusivity |
|  | **CCL2** | <-3 | 0.6 | Mutual exclusivity | <-3 | 0.54 | Mutual exclusivity |
|  | **CCL5** | <-3 | 0.6 | Mutual exclusivity | 0.11 | 0.611 | NS |
|  | **CCL17** | <-3 | 0.939 | Mutual exclusivity | <-3 | 0.91 | Mutual exclusivity |
|  | **CCL18** | <-3 | 0.83 | Mutual exclusivity | <-3 | 0.83 | Mutual exclusivity |
|  | **CCL22** | <-3 | 0.83 | Mutual exclusivity | <-3 | 0.91 | Mutual exclusivity |
|  | **TNF-α** | 1.67 | 0.09 | NS | 1.61 | 0.08 | NS |
|  | **IFN-γ** | <-3 | 0.78 | Mutual exclusivity | 0.1 | 1.51 | NS |

Odd ratio(OR) represents the co-occurrence of P2RY12 with molecules from the Supplementary Table. The significance was examined by Fisher’s exact testing.

Log(OR)>0, with P<0.05 indicates a trend of co-occurrence. Log(OR)<0 indicated the trend of mutual exclusivity.
