# Supplementary figures and images for: Expression site of P2RY12 in residential microglial cells in astrocytomas correlates with M1 and M2 marker expression and tumor grade
Source: Acta Neuropathol Commun. 2017 Jan 10;5:4. doi: 10.1186/s40478-016-0405-5 (PMC5223388; doi:10.1186/s40478-016-0405-5)

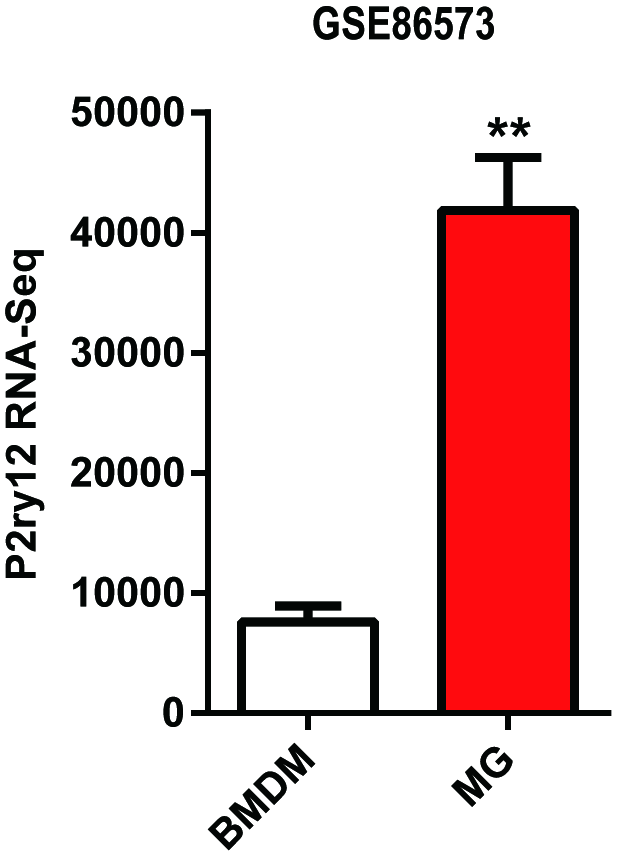

Supplement: Additional file 2: Figure S1. — Higher expression of P2ry12 in microglia than bone marrow derived macrophages in a mouse glioma model. It appears from the GEO dataset (GSE 86573) that P2ry12 mRNA is expressed by microglia (MG) and not by bone marrow-derived macrophages (BMDM) (Results are shown as Mean ± S.E.M representing the level of P2ry12 mRNA); ** P < 0.01. (TIF 2273 kb) [file 40478_2016_405_MOESM2_ESM.tif]

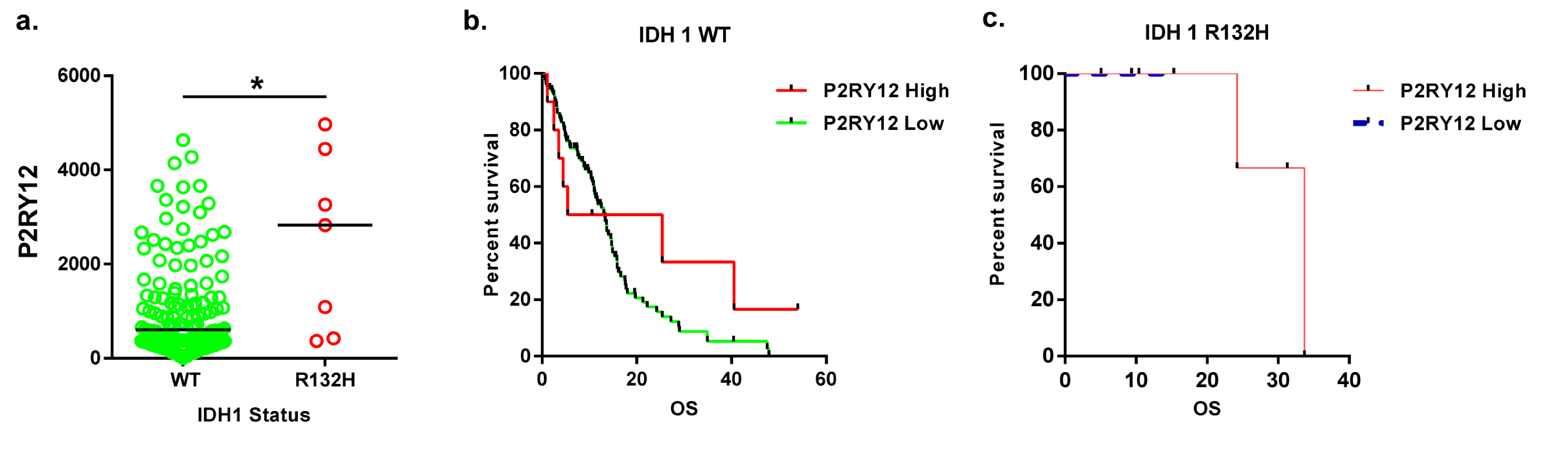

Supplement: Additional file 4: Figure S3. — Expression of P2RY12 does not influence overall survival in groups stratified for IDH1 mutation status (TCGA database). a. Increased P2RY12 mRNA expression in gliomas with IDH1 (R132H) mutation. Results are shown for the median values of P2RY12 mRNA levels; * P < 0.05; WT: IDH Wild type. b. Kaplan-Meier curves showing no effect of P2RY12 expression level on overall survival within the group of patients with IDH1 wild type gliomas (P > 0.1). c. Kaplan-Meier curves showing no effect of P2RY12 expression level on overall survival within the group of patients with IDH1 mutant (R132H) gliomas (P > 0.1). (TIF 10499 kb) [file 40478_2016_405_MOESM4_ESM.tif]

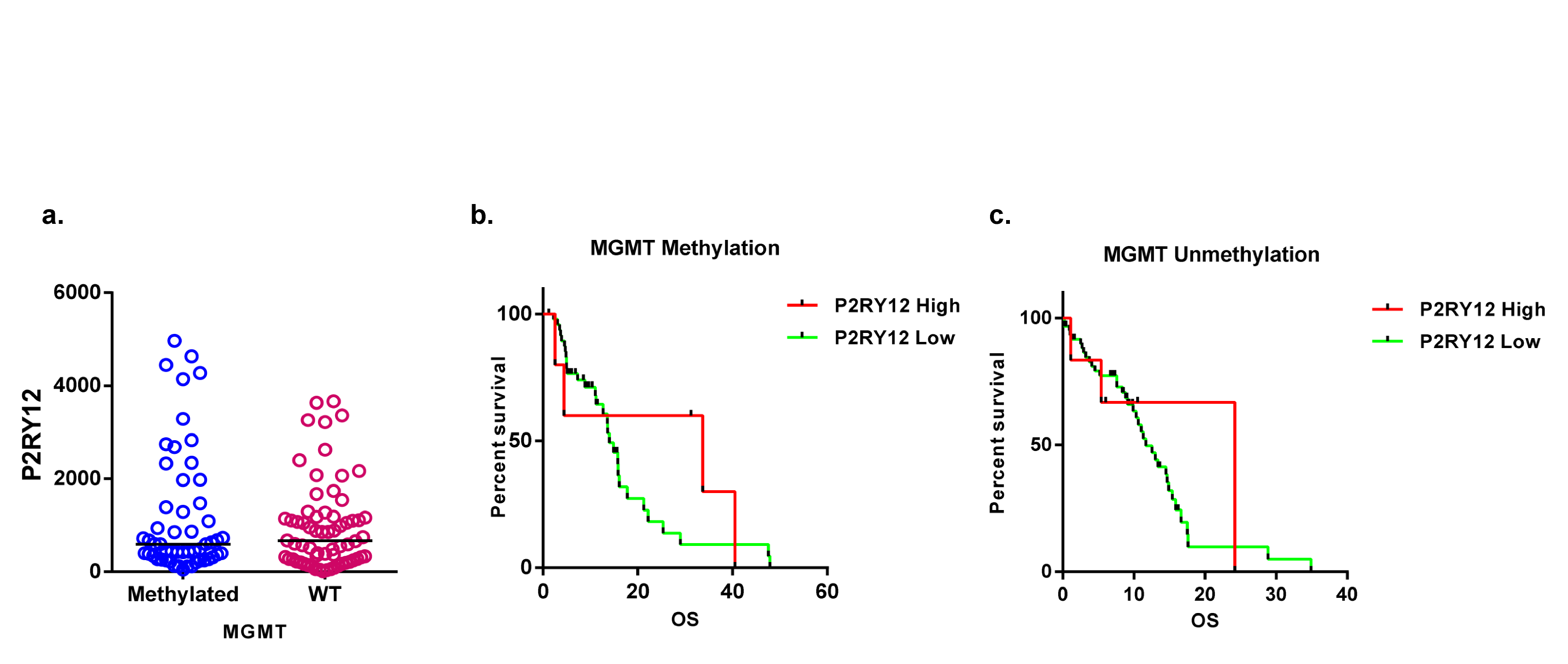

Supplement: Additional file 5: Figure S4. — P2RY12 does influence overall survival in gliomas stratified for MGMT promoter methylation status (TCGA database). a. P2RY12 mRNA expression levels in gliomas with and without MGMT promoter methylation (median values of P2RY12 mRNA levels are shown). b. Kaplan-Meier curves showing no influence of P2RY12 expression level on overall survival within the glioma group with methylated MGMT promoter. c. Kaplan-Meier curves showing no influence of P2RY12 expression level on overall survival within the glioma group with unmethylated MGMT promoter. (TIF 14397 kb) [file 40478_2016_405_MOESM5_ESM.tif]
